# The Impact of Training Dental Students to Use an Artificial Intelligence–Based Platform for Pulp Exposure Prediction Prior to Deep Caries Excavation: A Proof‐of‐Concept Randomised Controlled Trial

**DOI:** 10.1111/iej.70046

**Published:** 2025-10-10

**Authors:** Shaqayeq Ramezanzade, Tudor Laurentiu Dascalu, Azam Bakhshandeh, Sergio E. Uribe, Bulat Ibragimov, Lars Bjørndal

**Affiliations:** ^1^ Department of Cariology and Endodontics, Section for Clinical Oral Microbiology, Faculty of Health and Medical Sciences, Department of Odontology University of Copenhagen Copenhagen Denmark; ^2^ Department of Computer Science, Faculty of Science University of Copenhagen Copenhagen Denmark; ^3^ Department of Conservative Dentistry and Oral Health Riga Stradins University Riga Latvia; ^4^ Baltic Biomaterials Centre of Excellence Headquarters at Riga Technical University & RSU Institute of Stomatology Riga Latvia; ^5^ Department of Conservative Dentistry and Periodontology LMU University Hospital, LMU Munich Munich Germany

**Keywords:** artificial intelligence, dental caries, randomised controlled trial

## Abstract

**Aim:**

This study evaluated the effect of a short, personalised training session on student performance in using an artificial intelligence (AI)‐based platform for pulp exposure prediction before caries excavation and determined the required sample size for a further randomised controlled trial (RCT).

**Methodology:**

Undergraduate dental students were randomly assigned to the experimental (training) group and the control (no training) group. The training group received a 1‐h training session before undertaking the experiment, focusing on the uses, applications, and drawbacks of AI and carious lesion penetration depth. The theoretical presentation was followed by practical exercises and a quiz to check learning progress. Later, participants in both groups completed an experimental task involving 292 cases. They were asked to predict pulp exposure using an AI‐based website. Sample size calculations determined the required sample size, with 80% power and an alpha of 5%.

**Results:**

18 participants were enrolled (9 in each group). The agreement between participants' decisions and AI predictions regarding the presence or absence of pulp exposure (agreeableness with AI) was higher in the training group compared to the control group (0.83 vs. 0.76). The training group had a slightly higher mean F1‐score (0.63 vs. 0.62), accuracy (0.69 vs. 0.68), and sensitivity (0.63 vs. 0.59) than the control group. Based on the sample size calculation, at least 31 participants per group are needed for the future RCT.

**Conclusions:**

The results support further investigation of customised training sessions prior to using an AI‐based platform to assess their impact on dental students' agreement with AI predictions.

**Trial Registration:**

ClinicalTrial.gov identifier: NCT05912361

## 1 Introduction

Artificial Intelligence (AI) has shown significant potential in cariology and endodontics in image‐based diagnosis, designing treatment plans, and providing real‐time feedback to clinicians (Mohammad‐Rahimi et al. 2022; Ramezanzade, Laurentiu, et al. 2023). An increasing number of studies have evaluated the performance of AI compared with dental clinicians, demonstrating that AI often achieves accuracy comparable to that of dentists and sometimes significantly surpasses them (Ramezanzade, Laurentiu, et al. 2023; Khanagar et al. 2022). Few studies have assessed the ‘dentist‐AI collaboration’ (Li et al. 2022; Mertens et al. 2021; Ramezanzade, Dascalu, et al. 2023). Findings suggest that even when AI models performed well, not all dentists benefit from the AI‐generated recommendations (Mertens et al. 2021; Ramezanzade, Dascalu, et al. 2023). The AI integration into clinical workflows does not inherently enhance clinicians' performance. Among the factors preventing AI from fully integrating into clinical applications and realizing its maximum potential, one would be skepticism toward AI recommendations and insufficient knowledge about AI technology and its function (Cascella et al. 2023; Aristidou et al. 2022).

A fundamental understanding of AI is essential for successfully integrating its technology into routine practice and maximising its benefits (Ahuja 2019). Several frameworks have been suggested and implemented to incorporate AI in healthcare professionals' educational settings by offering courses in technology infrastructure, machine learning (ML), deep learning (DL), and data management alongside traditional biology courses (Islam et al. 2022; Turkkahraman 2023; Thurzo et al. 2023; Grunhut et al. 2021). One study emphasised the importance of radiologists' understanding of AI principles, research, and validation for effective AI applications (Slanetz et al. 2020). Clinicians have reported difficulty understanding AI literature, and they expressed a need for better training to understand and utilise AI. They preferred learning through real cases and questioned the reliability of AI algorithms in clinical practice (Slanetz et al. 2020). Moreover, the importance of introducing AI learning objectives as short courses in the medical curriculum has been discussed, focusing on its uses, applications, drawbacks, restrictions, and data management. Such training is intended to equip clinicians with the necessary skills for an AI‐augmented healthcare system (AlZaabi et al. 2023; Karaca et al. 2021). However, research on the effectiveness of short AI training courses for dental practitioners using AI‐based platforms remains limited.

A previously developed DL model helped to predict the risk of pulp exposure before deep caries excavation using clinical data about age and pain and two‐dimensional radiographs (Ramezanzade, Dascalu, et al. 2023). While two‐dimensional radiographs have inherent limitations in detecting pulp exposure, they are still commonly used to estimate risk in clinical and research contexts (Gasqui et al. 2022). Although the model performed well, participants did not fully benefit from its output, likely due to a limited understanding of how AI generates predictions. To explore whether training improves interpretation and trust, a proof‐of‐concept randomised controlled trial (RCT) was conducted. Given the novelty of this topic, effect size data were lacking, justifying the need for this preliminary study (Gluud 2006; Haynes et al. 1997).

The objective was to evaluate the effect of a short, personalised training session on student performance in using an AI‐based platform for pulp exposure prediction before caries excavation. Additionally, the effect size of training on diagnostic performance was estimated, and the sample size required to assess this effect in a future RCT was determined.

## 2 Materials and Methods

### 2.1 Protocol Registration and Ethical Considerations

The study followed the Preferred Reporting Items for Randomised Trials in Endodontics (PRIRATE) 2020 guidelines (Nagendrababu et al. 2020). This protocol was registered at ClinicalTrial.gov. Ethical approval was granted by the ethics committee of the related university (504‐0342/22‐5000), and data protection rules were followed (514‐0846/23‐3000) for using an AI‐based platform for predicting pulp exposure before deep caries excavation. The development of the AI model followed the checklist for AI in Dental Research (Schwendicke, Singh, et al. 2021).

### 2.2 Study Design

This study was designed as an RCT to evaluate the effect of training on the predictive performance of dental students in determining the binary outcome of pulp exposure versus no pulp exposure. The intervention consisted of a one‐hour training session covering the fundamentals and limitations of the AI system, along with hands‐on exercises using case‐based simulations. This was compared to a control group that received no training.

### 2.3 Dataset

A dataset of 292 cases with deep caries eligible for excavation therapy was used to develop the DL model. The cases were treated by dentists according to randomised allocation, either with stepwise excavation (*n* = 143) or non‐selective excavation (*n* = 149). Non‐selective excavation involved complete removal of carious dentine in one session, whereas stepwise excavation included selective excavation in the central area of the carious dentine performed in two stages, with the final restoration placed during the second visit. The results showed that pulp exposure occurred in 68 cases, 25 in the stepwise and 43 in the non‐selective group (Bjørndal et al. 2010).

### 2.4 The AI Model and the AI‐Based Platform

In previous research (Ramezanzade, Dascalu, et al. 2023), an automatic solution for predicting pulp exposure during caries excavation in teeth with extensive caries was developed. Data labeling and segmentation of deep caries, pulp, and cervical burnout were performed by Sh.R. Then, the distance between the pulp and the deepest part of the carious lesion was measured automatically. A pretrained DL model (ResNet 50) was trained and validated using the 10‐fold cross‐validation approach, achieving an accuracy of 0.78, F1‐score of 0.71, sensitivity of 0.62, specificity of 0.83, and area under the curve (AUC) of 0.73 (Ramezanzade, Dascalu, et al. 2023).

**FIGURE 1:**
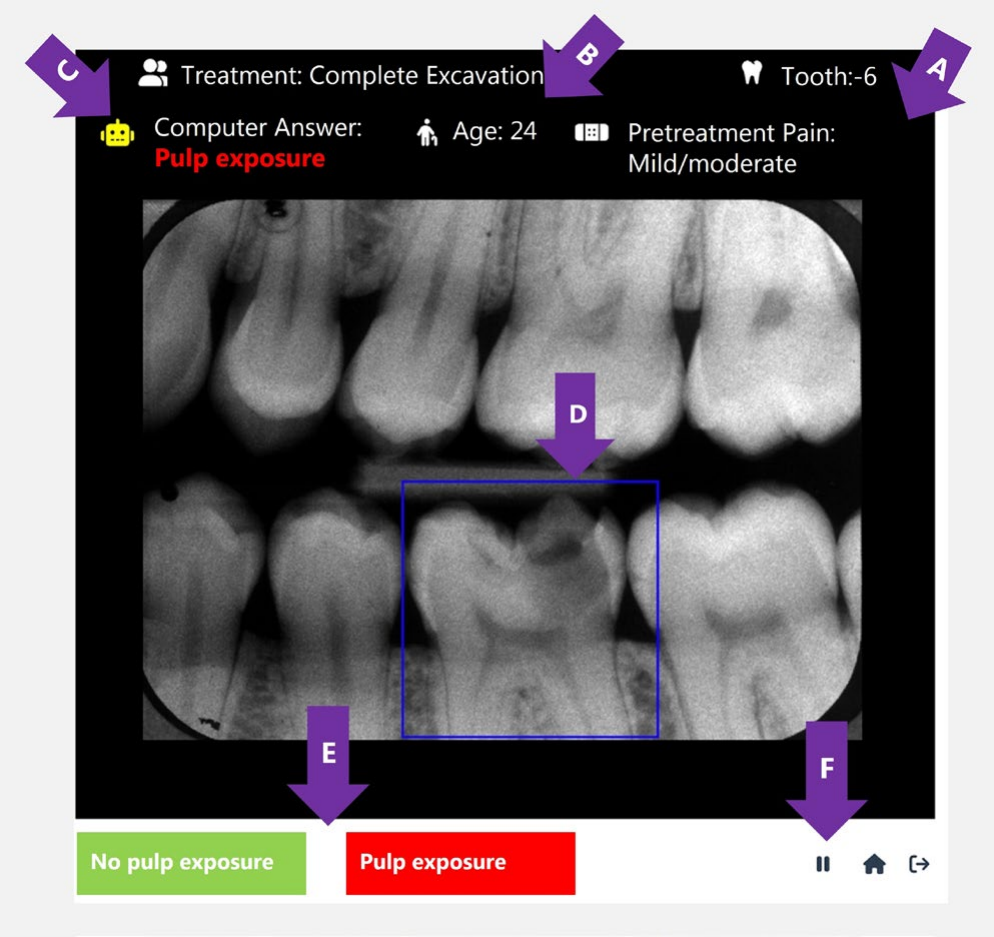
An illustration of the AI‐based platform for predicting the outcome of deep caries excavation. (A) Information about the tooth type, the presence of pretreatment pain, and the age of the patient. (B) The type of deep caries excavation treatment recommended for the case. (C) Computer prediction of pulp exposure based on the input data. (D) The tooth of interest, indicated by a square around it. (E) The two response options were provided to participants for their input. (F) The timing sign records response time and allowed for a 5‐min rest period if needed.

Subsequently, an AI‐based platform was designed where pretreatment radiographs along with the clinical patient information (age and pretreatment pain) were uploaded (Figure 1). Before including undergraduate participants, two senior dentists (L.B. and A.B.) took exercises, and the F1 score was lower than AI model (0.59 vs. 071).

### 2.5 Participants

The 25 participants from the previous study (Ramezanzade, Dascalu, et al. 2023), who were 4th and 5th‐year dental students with limited prior experience using an AI platform and knowledge of AI fundamentals and functionality, were invited to participate again after a minimum three‐month gap. All invited participants had similar levels of experience with AI and had completed the full curriculum covering basic and advanced courses in endodontics and cariology. However, the 5th‐year students had more clinical experience.

### 2.6 Randomization and Masking

A computerised randomization technique was used to allocate participants to the control and experimental groups in this RCT, following WHO guidelines. Random sequences were generated using validated software, with allocation concealment to prevent predictability, and rigorous documentation to ensure transparency. Participants were randomly assigned to either the experimental group (receiving training) or the control group (no training), stratified by gender and academic grade.

Participants were blinded to group allocation while blinding of personnel was not possible as the same administrator conducted the trial for both groups. The data analyst was blinded, as participants' responses on the AI‐based platform were stored in a pseudonymized manner.

### 2.7 Summary of the Setup

A single conductor (Sh.R.) with over 3 years of clinical experience and a profound comprehension of ML, DL, and AI oversaw the experiment for both groups. All tests were conducted individually, allowing the conductor to focus on one participant at a time. Participants who agreed to participate and provided informed consent were granted access to the experiment environment through the AI‐based platform.

### 2.8 Setup for the Control Group

In the control group, each participant watched a 5‐min video that provided details about the cases, including radiographic images, clinical data, treatment‐related data (the treatment type of stepwise versus complete excavation and each treatment's risk of pulp exposure), and AI predictions. The control group participants then had access to the AI‐based platform environment, which included options to choose between pulp exposure and no pulp exposure, a pause button, and a progress bar.

### 2.9 Setup for the Experimental Group

**FIGURE 2:**
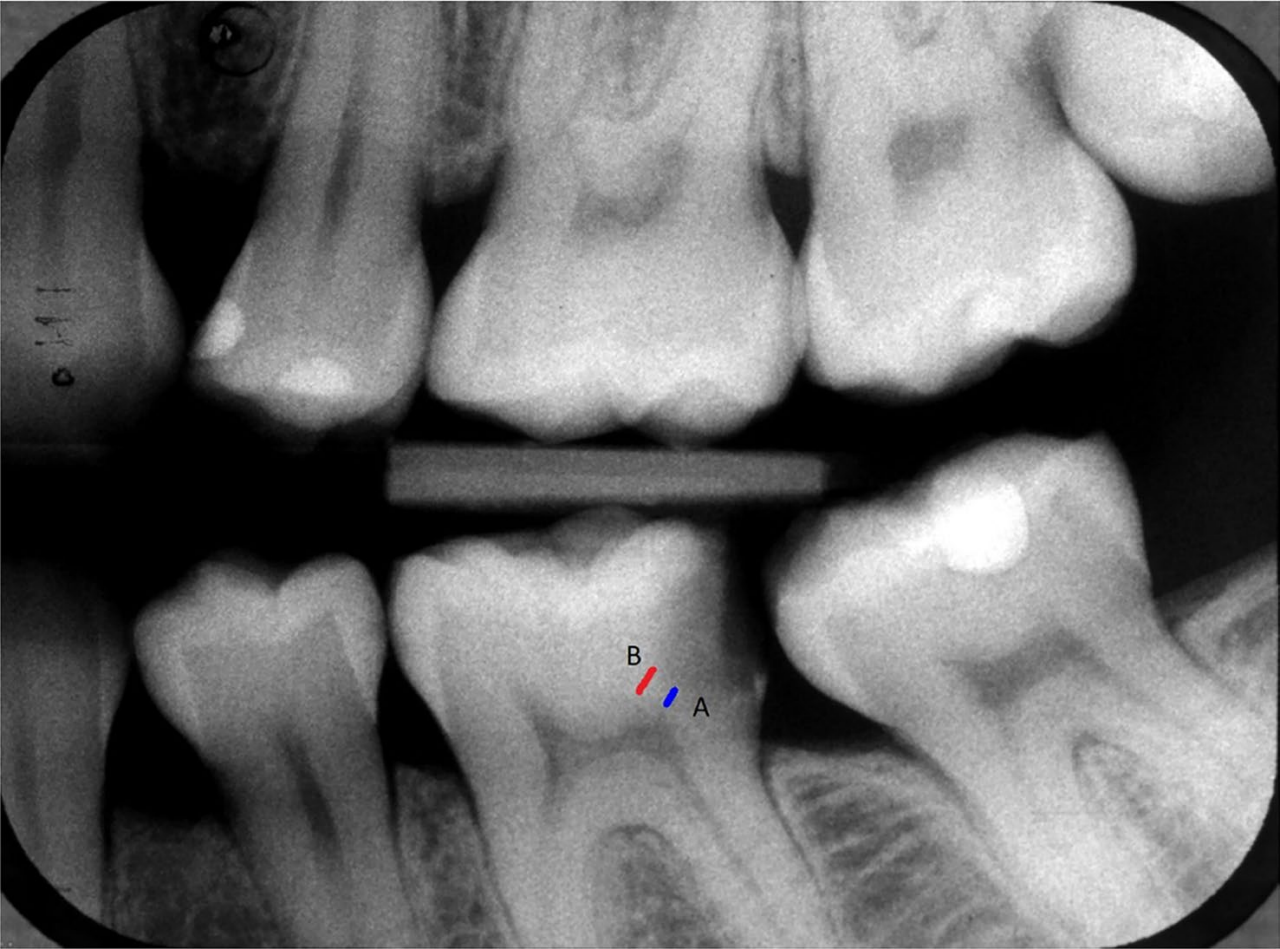
During the training of students in the experimental group, each participant underwent a hands‐on course involving 11 cases. In each case, the participant assessed a deep caries situation and selected the line that best illustrated the location of the shortest distance of the translucent zone reaching the pulp‐dentine border, reflecting penetration depth of carious dentine demineralization; in this case, A is the correct answer.

In contrast, the experimental group participants received a 1‐h training session before undertaking the experiment. During the training session, participants were exposed to the following key parts:

(i) In‐depth information about AI, including fundamental definitions of AI, ML, and DL. The training session also provided a detailed understanding of the different layers within Convolutional Neural Networks (CNNs). Additionally, there was a step‐by‐step explanation about training, validating, and testing the AI model used for outcome prediction in excavation therapy of deep caries. (ii) Reported accuracy and comparison with the accuracy of experienced dentists, the ability to find true cases of pulp exposure and cases without pulp exposure (as measured by sensitivity and specificity, respectively). (iii) Drawbacks of AI, particularly addressing the limitations of the current model in use, such as its black box nature. (iv) Treatment‐related data (the treatment type of stepwise versus complete excavation and each treatment's risk of pulp exposure) same as data given to the control group. (v) The participant would go through a hands‐on session of 11 cases on which the participant would check a case with deep caries and choose the line illustrating the shortest distance of the translucent zone reaching the pulp‐dentine border, reflecting penetration depth of carious dentine demineralization (Figure 2). The task of the participant was to find the line that best represented the distance between the variables described above. This exercise aimed to train participants' visual judgment on the correct border between the deepest penetration of carious dentine and the pulp wall border. After each answer, the participant's responses were discussed, and the correct answer was revealed, indicating the smallest distance between carious dentine and pulp as reflected by the radiograph. The students had access to the platform only if they answered correctly in at least 8 of 11 cases.

### 2.10 Metrics for Group Comparison and Sample Size Calculation

The primary outcome measure was agreeableness with AI, defined as the degree of alignment between participants' decisions and AI‐generated predictions. For each case on the AI‐based platform, if the participant's answer matched AI's prediction, the agreeableness with AI was recorded as 1, and 0 in the case of disagreement. An individual agreeableness was calculated for each participant, and the mean value was used for sample size estimation. The secondary outcomes included accuracy (Acc), F1‐score, sensitivity (Sen), and specificity (Spec). In addition, the response time for each participant for each question was recorded in both groups.

### 2.11 Statistical Methods

**FIGURE 3:**
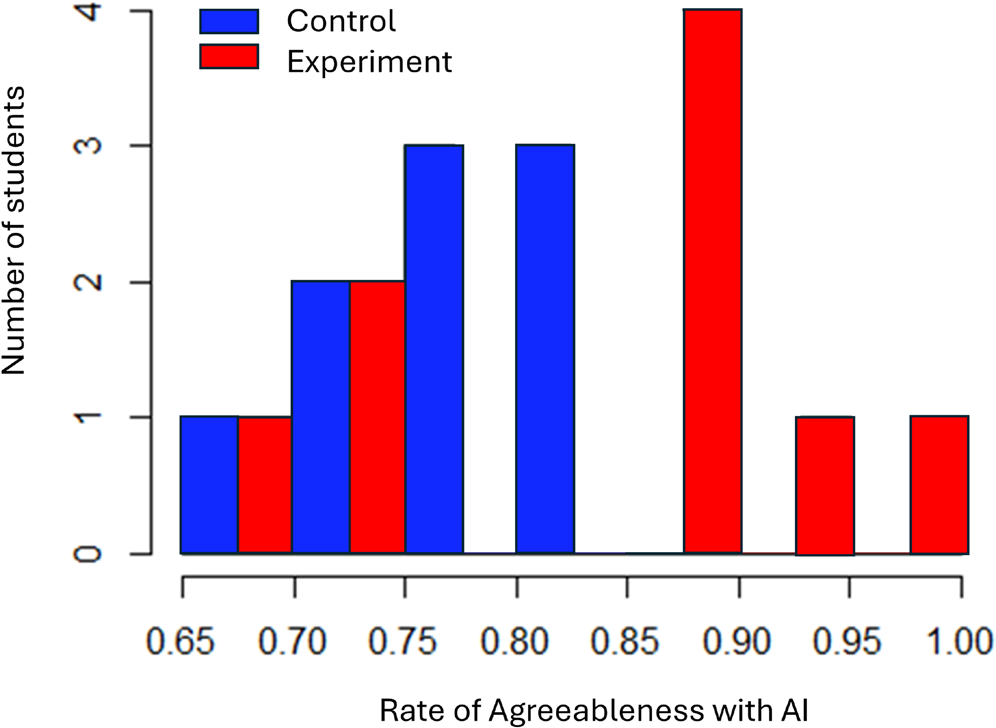
The histogram for agreeableness with AI in experimental and control groups (each unit represents one participant).

The distribution of agreeableness of participants with AI was examined using histograms (Figure 3). The 95% confidence intervals for the mean of agreeableness scores with AI in experimental and control groups were calculated. Hedges' g (a modified version of Cohen's d that accounts for small sample sizes) was calculated for agreeableness with AI. Then, sample size calculation was conducted based on the observed effect size, using a significance level of *α* = 0.05, a 95% confidence interval (CI), and 80% power (*β* = 0.20, Type II error).

### 2.12 Criteria for Progression to Full Trial

The decision to move to a full RCT will be based on several key criteria from this proof of concept:

- Recruitment rate: At least 70% of the target sample must be recruited within the designated period.
- Adherence to protocol: At least 80% of participants must complete the intervention and follow‐up to ensure adequate adherence.
- AI predictions agreement: The effect size for agreement between the experimental and control groups should be at least 0.5, indicating a moderate effect.

## 3 Results

**FIGURE 4:**
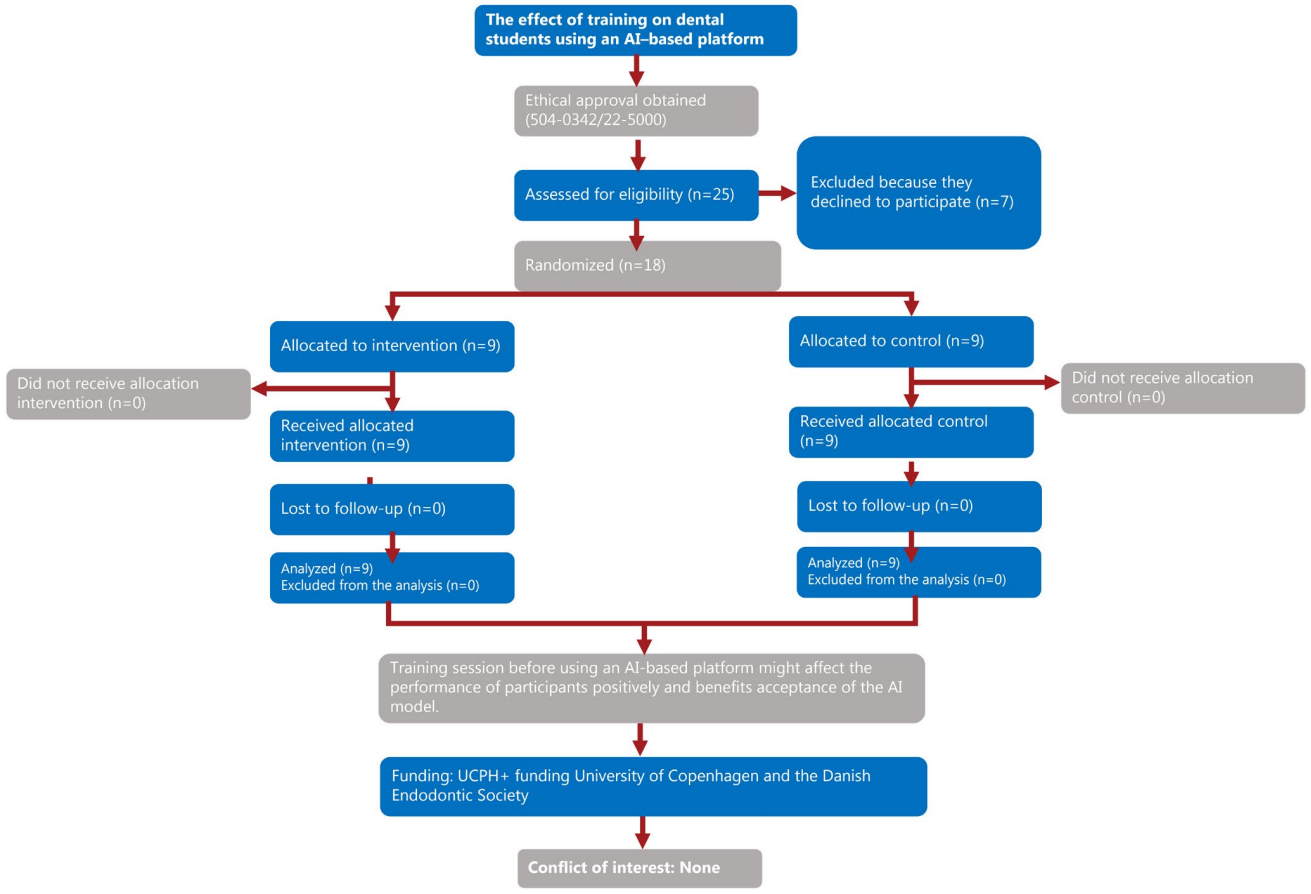
The PRIRATE 2020 flowchart illustrates the participant flow throughout the trial.

Out of 25 invited participants (Ramezanzade, Dascalu, et al. 2023), 18 participants agreed to participate again in this study (15 female and 3 male students); while 13 participants were 5th‐year dental students, 5 were 4th‐year dental students. Participants were randomly assigned to experimental or control groups. The PRIRATE 2020 flowchart illustrates the participant flow throughout the trial (Figure 4).

All participants in the experimental group answered at least 8 out of 11 cases in the hands‐on practice quiz, with a mean of 9.5 correct answers.

**FIGURE 5:**
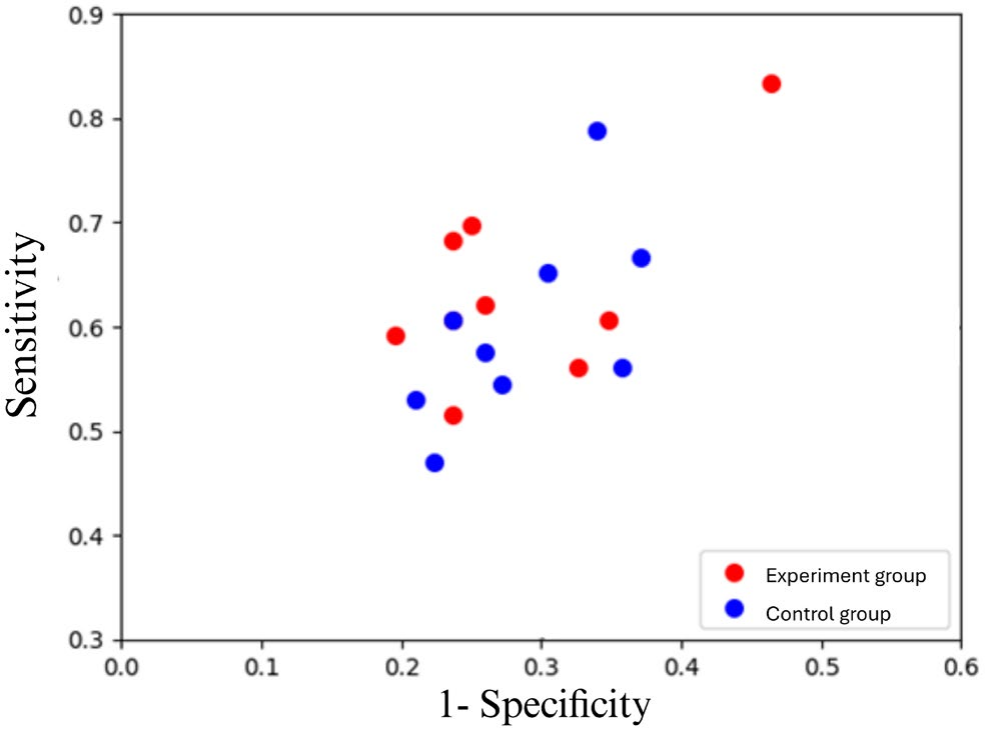
The scatterplots of all participants in the experiment and control group.

The agreeableness with AI in the experimental group was higher than in the control group (0.83 vs. 0.76). Participants in the experimental group had slightly higher mean F1‐score and accuracy than the control group (0.63 vs. 0.62 and 0.69 vs. 0.68, respectively). The experimental group showed higher sensitivity (0.63) than the control group (0.59). There was no difference in specificity between the two groups. The experimental group expended slightly lower time (12.27 s) answering questions than the control group (14.12 s). The scatterplots of all experimental and control group participants were plotted to allow for visual assessment (Figure 5). The results are summarised in Table 1. The histogram for agreeableness with AI as the main metric is illustrated in Figure 3. The 95% CI for agreeableness with AI in the experimental group was 0.75–0.90 and 0.72–0.81 for the control group. Additionally, the two‐sample *t*‐test did not reveal a significant difference in agreement with AI between the experimental and control groups. The effect size (Hedges' *g*) was 0.72. The power calculations based on the results achieved in the present study indicated that a minimum of 31 participants in each group would be needed for the next RCT to test the hypothesis.

**TABLE 1:** Mean and 95% confidence interval values for participants' performance in the experimental and control groups.

	Experimental	Control
Agreeableness with AI answers	0.83 (0.76–0.90)	0.76 (0.72–0.80)
F1‐score	0.63 (0.60–0.66)	0.62 (0.60–0.64)
Accuracy	0.69 (0.65–0.73)	0.68 (0.66–0.71)
Sensitivity	0.63 (0.60–0.64)	0.59 (0.53–0.66)
Specificity	0.71 (0.65–0.77)	0.71 (0.67–0.75)
Time for answering questions (Sec)	12.27 (10.93–13.61)	14.12 (11.77–16.46)

## 4 Discussion

AI in dentistry, as an educational tool, is still a ‘work in progress’. Although several FDA‐cleared AI software products exist and research in the dental field is increasing (Mertens et al. 2021; Axis Imaging News 2024; Issa et al. 2023), clinicians and dental students lack sufficient insight into the strengths and potential pitfalls associated with AI (Gowda et al. 2022).

Several studies have attempted to predict pulp exposure in deep caries excavation with promising results. A study (Wang et al. 2023) used a dataset of 206 radiographs from teeth with caries and pulpitis to develop a CNN (DenseNet) capable of predicting pulp exposure during caries excavation therapy, achieving an AUC of 0.97. Another study utilized ResNet18 to differentiate between radiographically deep and extremely deep caries, demonstrating an AUC of 0.94 on 844 radiographs (Zheng et al. 2021). Ramezanzade, Dascalu, et al. (2023) assessed dentist‐AI interaction using their pulp exposure prediction model (a multipath CNN based on ResNet50), which achieved a macro F1‐score of 0.71. However, the benefits of AI assistance were relatively limited, raising the performance of the participants from 0.59 to 0.61 in terms of macro F1‐score, which remains significantly lower than the performance achievable if participants had placed complete trust in AI. Recent studies of AI‐based diagnostic systems have shown that even when these tools provide accurate assessments, they may not improve patient outcomes if clinicians do not incorporate the information into their diagnostic or treatment decisions (Schwendicke, Rossi, et al. 2021). Similarly, in medical research, large language models (LLMs) have been used to assist clinicians for diagnostic reasoning and showed that clinicians often undervalue AI input in favor of their own judgment, and providing access to LLMs alone did not significantly improve physicians' diagnostic reasoning. To address this, it is essential to train clinicians in effective AI use, integrate AI tools into clinical workflows, and design systems that support reflective thinking by presenting both supporting and opposing diagnostic evidence (Agarwal et al. 2023; Goh et al. 2024).

Similarly, a study involving 324 chest X‐rays, 227 radiologists, and over 40 000 diagnostic decisions was done to assess radiologist‐AI collaboration. While AI outperformed 78% of radiologists in diagnostic accuracy (based on ROC AUC), AI assistance alone did not improve average human performance. In fact, the radiologist did not appreciate the AI assistance benefits due to skepticism and error anticipation in AI predictions (Moehring et al. 2025; Agarwal et al. 2023).

This disconnect emphasizes the importance of evaluating how clinicians interact with AI tools. Our study addresses this issue by investigating whether structured training can influence decision‐making, shifting the focus from model accuracy to potential patient benefit.

In this study, the experimental group demonstrated a higher mean level of agreeableness with AI compared to the control group. Also, based on Table 1, there were borderline improvements in some performance metrics such as F1‐score, accuracy, and sensitivity within the experimental group versus the control group. The overlapping CIs indicate the need for more data to draw conclusions. The observed effect size for the difference in agreeableness with AI between the two groups was 0.72, indicating a considerable effect of the intervention. This suggests that the training program had a measurable impact on participants' ability to align with AI predictions. To further validate these findings, a larger, adequately powered randomised controlled trial (RCT) is warranted to rigorously test our null hypothesis. Based on power calculations, at least 31 participants per group are needed to achieve 80% power to detect a statistically significant difference in agreeableness between the experimental and control groups, assuming a significance level of *p* < 0.05. This sample size would allow for a more robust evaluation of the training intervention's effect on AI‐assisted decision‐making and secondary metrics such as accuracy, F1‐score, and sensitivity.

Given the effect size observed in this study, future trials are needed to fully assess the impact of AI training on clinical decision‐making metrics and to confirm the feasibility of implementing AI platforms in routine dental practice.

The topics presented to participants primarily focused on AI, ML, and DL fundamentals. Specifically, the training session covered the processes involved in training, testing, and interpreting the model. During this training, participants gained insights into the decision‐making logic employed by the model. This understanding is crucial, as it helps address the opacity of AI models, often referred to as the ‘black box’ problem, where the reasoning behind an algorithm's output is not easily interpretable.

A personalised one‐on‐one training session was advocated to allow participants to learn at their own pace. It also accommodated participants' differences, such as learning styles, prior knowledge, preferences, and ability levels (Chen and Wang 2021). Furthermore, a hands‐on training session was conducted before the main experiment. The hands‐on session was designed to demonstrate how the AI determines the penetration depth of the carious lesion (based on the line that it draws between the advancing front of the radiolucent zone reflecting carious dentine and the closest part to the border of the pulp chamber). It would also train participants to focus their attention on this particular region. However, on a larger scale, this method may be costly and challenging. Previous studies have explored personalised training approaches that address individual needs and provide immediate, on‐demand support. Examples include educational chatbots and software platforms that track users' behaviour to suggest relevant video lectures for training radiologists (Fang et al. 2024; Gokli et al. 2021). Considering that a dentist's knowledge of AI may influence the benefit of an AI platform, the next step is to explore better integration methods.

The experimental group reported higher mean sensitivity than the control group. However, the specificity was the same, meaning that the experimental group was better at finding cases that would end up in pulp exposure. Still, their ability to correctly identify cases without pulp exposure was the same with and without training. This might suggest that the training session could train participants' eyes in finding the subtle signs or locations where the line between deepest carious dentine penetration (as evidenced by the radiolucent zone penetration) and the pulp‐dentine border may be intermittent, potentially leading to pulp exposure. Conversely, the training session had no impact on the participants' ability to accurately identify healthy teeth in this AI‐based platform, which seems logical as there was no particular extra training involving the signs of healthy teeth. Previous research also confirms this, as variations in sensitivity among different groups of dental professionals with different expertise have been observed in caries detection, while specificity tends to remain consistent across dentists with different experiences (Sato et al. 2021).

The agreeableness with AI was higher in the training group compared with the control group (0.83 vs. 0.76), suggesting that the training session positively influenced their trust in the model. On the other hand, the answering time was slightly faster in the experimental group (12.27 s) compared to the control group (14.12 s). The increased agreeableness and slightly faster responses in the experimental group suggest that the training session improves students' trust in AI. Participants appeared to make more efficient and reasoned decisions without developing ‘blind trust’, thus avoiding automation bias while still effectively using AI.

While this study primarily focused on technical metrics like agreeableness with AI, accuracy, and sensitivity under controlled conditions, future trials should explore the practical implications of AI‐assisted decision‐making in real‐world patient care. For example, evaluating the proportion of patients who avoid unnecessary restorative treatments thanks to AI predictions could provide critical insights into its clinical utility. Additionally, examining the societal impact, such as reduced healthcare costs and improved access to care through AI integration, would further support its broader implementation in dental practices.

A greater level of agreeableness with AI among participants in the experimental group implies that they were more inclined to follow the AI's predictions than the control group (0.83 vs. 0.76). Nevertheless, the comparable accuracy (0.69 vs. 0.68) and F1‐score (0.63 vs. 0.62) at the experimental and control groups suggest that, despite this increased agreeableness, their practical performance in accurately predicting outcomes did not change notably. This may be because aligning more closely with AI does not inherently improve accuracy or F1‐score as both participants and the AI may have answered wrongly in challenging cases. Also, there might be differences in the participants' contemporary understanding of advanced caries compared to the AI model, as the model has been trained on the cases and treatment outcomes collected several years ago. If a more robust model had been used, for example, one with an F1 score of 90%, the effect of training might have been more evident in traditional performance metrics, as following AI guidance from a highly accurate model would likely lead to improved outcomes. Therefore, ‘Agreement with AI’ was introduced as a new metric, which is less dependent on the AI model's baseline performance and provides a more reliable measure of how training influences students' trust in and alignment with AI recommendations. Despite efforts to blind participants to group allocation, individuals were inevitably aware of whether they received the training. Combined with the limited sample size, the results should be considered reliable only within this study setup.

## 5 Conclusion

A short, personalised training session before using an AI‐based platform may have improved students' agreement with AI. These results support further investigation of customised training sessions to assess their impact on dental students' agreement with AI predictions. A larger, multicenter trial is needed to determine whether training enhances the effective use of AI for pulp exposure prediction before deep caries removal.

## Author Contributions

S. Ramezanzade: conceptualization, methodology, data curation, formal analysis, writing – original draft preparation. T. L. Dascalu: conceptualization, methodology, formal analysis, writing – original draft preparation. A. Bakhshandeh: conceptualization, methodology, validation, supervision, writing – review and editing. S. E. Uribe: methodology, validation, supervision, writing – review and editing. B. Ibragimov: conceptualization, methodology, validation, supervision, writing – review and editing. L. Bjørndal: Conceptualization, methodology, validation, supervision, writing – review and editing.

## Ethics Statement

This protocol was registered at ClinicalTrial.gov. Ethical approval was granted by the University of Copenhagen (504‐0342/22‐5000), and data protection rules were followed (514‐0846/23‐3000).

## Consent

The authors have nothing to report.

## Conflicts of Interest

The authors declare no conflicts of interest.

## Data Availability

The anonymous data about participants' responses are available in the supplementary material hosted on Zenodo (https://doi.org/10.5281/zenodo.15209174). The codes for the model are available at the following link: https://github.com/tudordascalu/pulp‐exposure‐classification.
